# Developing high-concentration monoclonal antibody formulations for subcutaneous administration to improve patient treatment

**DOI:** 10.1007/s12551-025-01346-2

**Published:** 2025-08-02

**Authors:** Laura R. R. Mijangos, Stephen E. Harding, Nicholas J. Darton

**Affiliations:** 1 National Centre for Macromolecular Hydrodynamics, School of Biosciences, University of Nottingham, Sutton Bonington, UK; 2 Dosage Form Design and Development, AstraZeneca, BioPharmaceuticals R&D, 1 Francis Crick Avenue, Cambridge, Cambridgeshire CB2 0AA UK

**Keywords:** High-concentration monoclonal antibody products, Oncology, High viscosity, Oncology, Formulation, Excipients

## Abstract

The transition of immunotherapy administration from intravenous infusion to subcutaneous (SC) administration of monoclonal antibody formulations for oncology patients has garnered significant interest. SC administration offers multiple benefits, including potential for at-home administration, enhanced patient compliance, reduced hospital congestion, lowered health care costs, and improved sustainability by reducing drug wastage and minimizing environmental impact. However, for many biologics, the shift to SC administration requires the development of high-concentration monoclonal antibody products (HCmAP) due to the need for large dose volumes. Here we explore the impact of the COVID-19 pandemic on immunotherapy administration and the imperative of adopting SC administration. We discuss challenges encountered throughout the manufacturing, shipping, storage, and delivery of HCmAP. A central hurdle identified involves the biophysical instability and the large increase in viscosity of these biologics due to increased antibody concentration. Further complications can arise from “non-ideality” effects through molecular crowding or co-exclusion effects (macromolecules blocking the free movement in solution of other macromolecules) and elevated macromolecular interactions. For reducing the viscosity for a given concentration of antibody, the main excipients reported are salts and amino acids, with Arg-HCl demonstrating particularly improved formulation viscosity in an HCmAP. However, excipients with viscosity-lowering effects can also impact protein stability. The journey to discover suitable excipient strategies remains ongoing, combined with emerging approaches such as molecular engineering and computational techniques, with the ultimate aim of facilitating the successful integration of SC administration for economic savings, environmental sustainability, and social equity.

## Introduction

As of 2023, the U.S. Food and Drug Administration (FDA (U.S. n.d).) had approved a total of 168 monoclonal antibodies (mAbs) for a wide range of therapeutic areas (U.S. Food and Drug Administration (U.S. n.d.)). Approximately 43% of these approved mAbs are for oncologic indications, including solid tumors, lymphomas, multiple myelomas, and leukemia, followed by about 23% for immunologic indications, such as rheumatoid arthritis and systemic lupus erythematosus. Almost 9% of approvals are for neurologic indications. This is followed by hematology and respiratory diseases, each comprising almost 5%. Infectious diseases, ophthalmology, and gastroenterology each represent 3.5–4%. Finally, cardiology and endocrinology each represent 2% of mAb approvals.

**Fig. 1:**
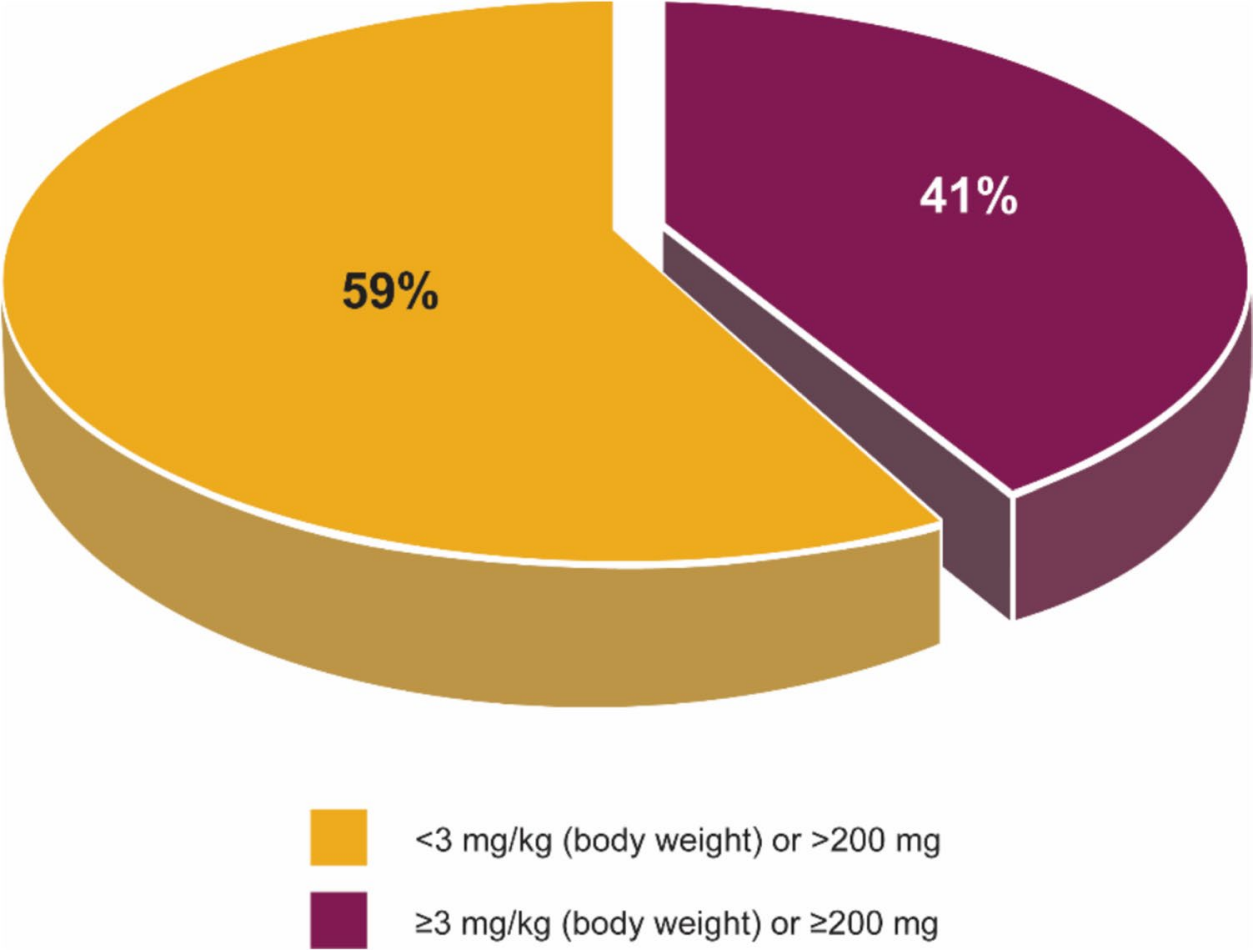
Recommended dose distribution of FDA-approved mAbs across therapeutic areas. The majority of mAb products are prescribed with a minimum dose of 200 mg or higher. The available evidence consistently indicates a notable trend toward higher dose requirements in mAb therapy

**Fig. 2:**
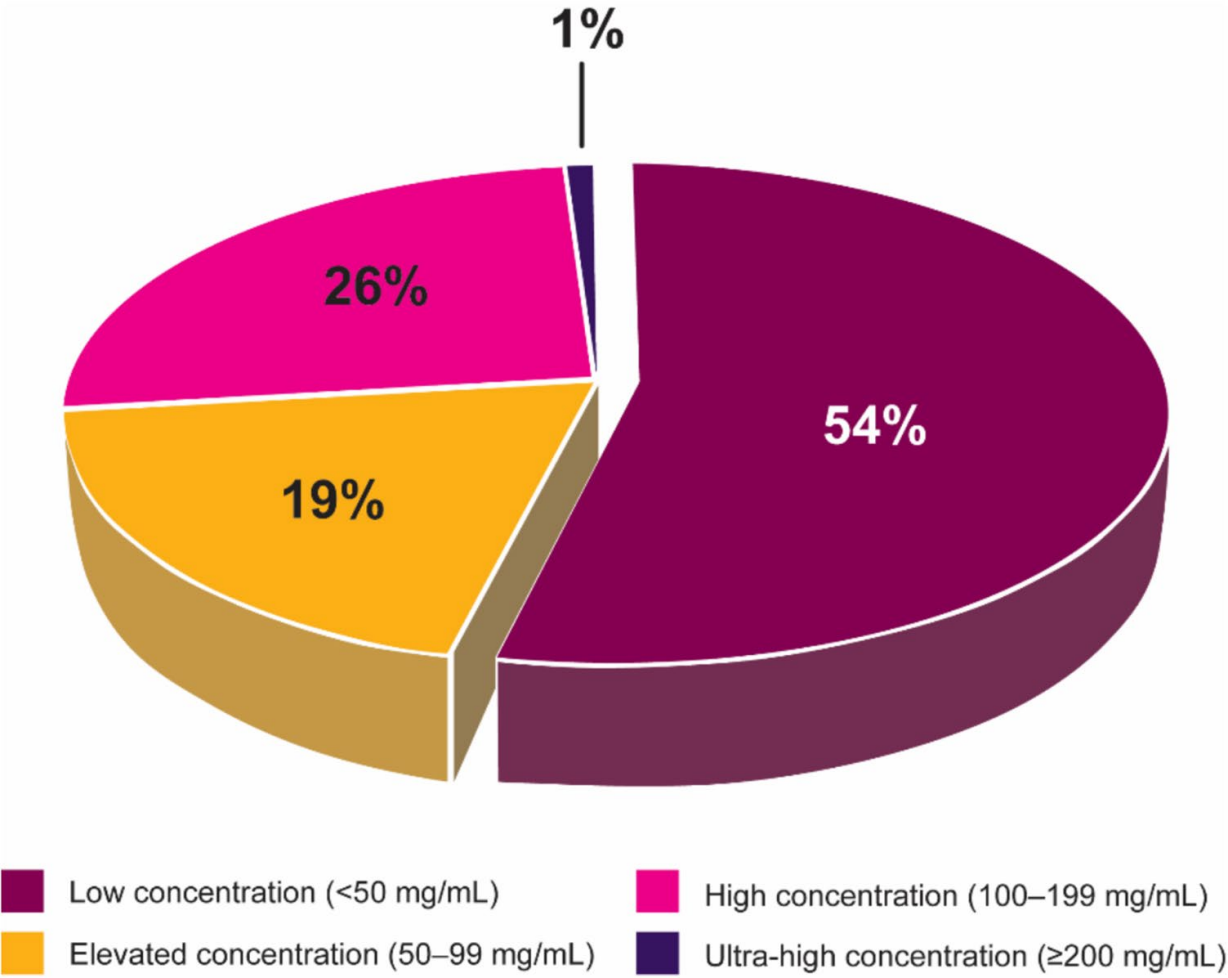
Protein concentration of FDA-approved mAb formulations. The majority of currently available mAbs are provided in low concentrations. However, there has been a notable increase in the production of HCmAP

**Fig. 3:**
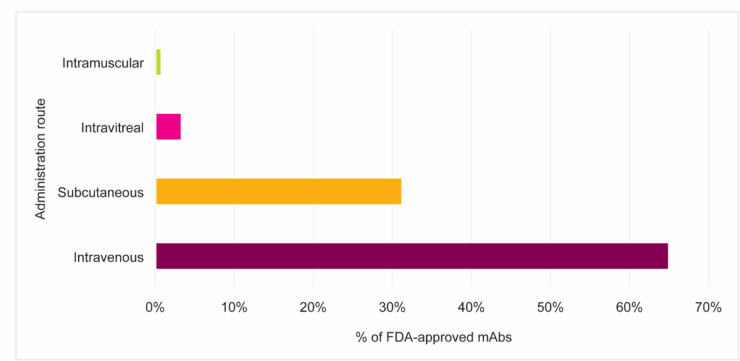
Distribution of administration routes of FDA-approved mAbs. IV infusion is the most commonly used route for mAb therapy, followed by SC administration

Treatment with a mAb therapy often requires high doses to achieve therapeutic effects. Almost 60% of FDA-approved mAbs are prescribed in a minimum dose of 200 mg per administration (Fig. 1) and require high-concentration formulations and/or large volumes. Because 54% of FDA-approved mAbs are produced at relatively low concentrations (< 50 mg/mL) (Fig. 2), a high volume is required to achieve these high doses. Due to these requirements, the prevalence of lower-concentration formulations, and the need for large solution volumes, intravenous (IV) administration is often the most feasible administration route (Fig. 3). The IV route offers the advantage of delivering the drug directly into the systemic circulation, ensuring high bioavailability. However, the invasive nature of IV therapy (Bittner et al. 2018) can be associated with potential complications such as phlebitis, infiltration, extravasation, and infections (Dychter et al. 2012).

A notable trend emerges when comparing administered doses in immunology and oncology. Approximately 53% of mAbs in immunology require a dose of ≤ 200 mg, whereas in oncology, only about 30% are prescribed at ≤ 200 mg and 70% require a dose of ˃200 mg. This contrast presents considerable challenges due to the necessity for larger injection volumes or multiple injections to administer the required dose. Consequently, the development of high-concentration mAb products (HCmAP) is required to bridge this gap, enabling the administration of high doses in small volumes to facilitate the transition from IV to subcutaneous (SC) administration.

Switching from IV to SC administration offers multiple benefits for hospitals, patients, and health care professionals. From a medical perspective, it reduces congestion in medical facilities, allowing priority to be given to patients in need of emergency treatment, as occurred during the COVID-19 pandemic. In addition, it helps minimize adverse events associated with IV infusion. Patients also prefer a more convenient and easily administered treatment. The possibility of supervised at-home administration provides them with a flexible treatment schedule, thereby reducing travel expenses to hospitals and lowering the risk of hospital-acquired infections. Additional benefits include decreased health care costs (Jonaitis et al. 2021). Dychter et al. (2012) conducted an economic evaluation to assess the cost-effectiveness of IV therapy. They demonstrated that IV delivery is significantly more expensive than SC delivery of the same biologic. For example, the average cost for nursing labor to administer an SC dose of alemtuzumab was $30, whereas an IV infusion of alemtuzumab cost an average of $113.

Although SC administration offers numerous advantages, a number of potential challenges and drawbacks are associated with this route of administration. These may include lower bioavailability due to slower absorption from SC tissue. Another challenge is posed by the presence of hyaluronan and collagen fibres in the SC connective tissue, which act as barriers that impede the spread of injected fluids and hinder large-volume injections. It is important to stress that SC, although providing the basis for a more convenient form of administration, brings in new issues caused by high-concentration, namely, “non-ideality” effects through molecular crowding and/or co-exclusion effects [macromolecules blocking the free movement in solution of other macromolecules; see, e.g., Harding (1997)] and elevated self-associative or aggregative interactions, all factors contributing to lower efficacy of formulations.

The focus of this review is threefold:

1. **Assessment of HCmAP:** We evaluate the necessity for the development of HCmAP to facilitate SC administration and examine the impact of the COVID-19 pandemic on the administration of immunotherapy among oncology patients.
2. **Manufacturing, storage, and administration challenges:** A detailed analysis is conducted to identify the challenges associated with the manufacturing, storage, and administration of HCmAP.
3. **Formulation strategies:** We explore potential strategies for the formulation of HCmAP, focusing on the use of various excipients to enhance their stability.

## Formulation of HCmAP

The transition from IV infusion to SC administration requires the development of high-concentration formulations, defined here as injectable mAb therapies with an overall product concentration of 100–200 mg/mL. Currently, there are 45 HCmAP on the market (Table 1). Of these formulations, 17 are prescribed primarily in the field of immunology and only 4 are approved for oncologic indications. The dominance of HCmAP in the field of immunology is to be expected, considering the chronicity of these diseases and the need for long-term management aimed at enhancing the quality of life for patients.

**Table 1:** HCmAP approved by the FDA (U.S. Food and Drug Administration (U.S. n.d.))

Name	Active ingredient	Company	Concentration	Dose	Route	Antigen	Indication(s)	Initial U.S. approval (year)
Synagis	palivizumab	Sobi	100 mg/mL	15 mg/kg	IM	RSV F protein inhibitor	Lower respiratory tract disease caused by RSV	1998
Xolair	omalizumab	Genentech	125 mg/mL	150 and 300 mg	SC	Anti-IgE	Asthma, chronic idiopathic urticaria	2003
Susvimo	ranibizumab	Genentech	100 mg/mL	2 mg	Intravitreal	VEGF	Neovascular (wet) age-related macular degeneration	2006
Cimzia	certolizumab pegol	UCB	200 mg/mL	400 mg	SC	TNF	Crohn’s disease, rheumatoid arthritis, psoriatic arthritis, ankylosing spondylitis	2008
Ilaris	canakinumab	Novartis	150 mg/mL	150 mg	SC	IL-1β	Periodic fever syndromes, systemic juvenile idiopathic arthritis	2009
Simponi	golimumab	Centocor	100 mg/mL	50 mg	SC	TNF	Rheumatoid arthritis, psoriatic arthritis, ankylosing spondylitis	2009
Actemra	tocilizumab	Genentech	20 mg/mL	4, 10, 8, and 12 mg/kg	IV	IL-6	Rheumatoid arthritis, giant cell arteritis, polyarticular juvenile idiopathic arthritis, systemic juvenile idiopathic arthritis, cytokine release syndrome	2010
			180 mg/mL	162 mg	SC			
Nucala	mepolizumab	GlaxoSmithKline	100 mg/mL	100 mg	SC	IL-5	Severe asthma, chronic rhinosinusitis with nasal polyposis, eosinophilic granulomatosis with polyangiitis, hypereosinophilic syndrome	2015
Praluent	alirocumab	Regeneron	75 and 150 mg/mL	75 mg	SC	PCSK9	Heterozygous familial hypercholesterolemia, clinical atherosclerotic cardiovascular disease	2015
Repatha	evolocumab	Amgen	140 and 120 mg/mL	140 and 420 mg	SC	PCSK9	Risk of myocardial infarction, hyperlipidemia; homozygous familial hypercholesterolemia	2015
Anthim	obiltoxaximab	Elusys Therapeutics	100 mg/mL	16, 24, and 32 mg/kg	IV	*Bacillus anthracis*	Inhalational anthrax	2016
Cosentyx	secukinumab	Novartis	150 mg/mL	300 and 150 mg	SC	IL-17A	Severe asthma	2016
Zinbryta	daclizumab	Biogen	150 mg/mL	150 mg	SC	IL-2	Multiple sclerosis	2016
Dupixent	dupilumab	Regeneron	150 and 175 mg/mL	600 and 400 mg	SC	IL-4	Atopic dermatitis, asthma, chronic rhinosinusitis with nasal polyposis, eosinophilic esophagitis	2017
Hemlibra	emicizumab-kxwh	Genentech	30 and 150 mg/mL	3 and 1.5 mg/kg	SC	Factor IXa and factor X	Hemophilia A	2017
Kevzara	sarilumab	Sanofi	131 and 175 mg/mL	200 mg	SC	IL-6	Rheumatoid arthritis	2017
Rituxan hycela	rituximab, hyaluronidase (human recombinant)	Genentech	120 mg, 2000 U/mL	1400 mg rituximab/23,400 U hyaluronidase human, 1600 mg/26,800 U	SC	CD20	Follicular lymphoma, diffuse large B-cell lymphoma, chronic lymphocytic leukemia	2017
Siliq	brodalumab	Valeant	140 mg/mL	210 mg	SC	IL-17RA	Plaque psoriasis	2017
Tremfya	guselkumab	Janssen	100 mg/mL	100 mg	SC	IL-23	Plaque psoriasis, psoriatic arthritis	2017
Ajovy	fremanezumab-vfrm	Teva	150 mg/mL	225 and 675 mg	SC	Calcitonin	Migraine	2018
Emgality	galcanezumab-gnlm	Eli Lilly	120 mg/mL	240 and 120 mg	SC	Calcitonin	Migraine	2018
Herceptin hylecta	Trastuzumab, hyaluronidase-oysk	Genentech	120 mg/2000 U/mL	600 mg and 10,000 U hyaluronidase per 5 mL	SC	HER2	HER2-overexpressing breast cancer	2018
Humira	adalimumab	Abbvie	100 and 50 mg/mL	80 and 40 mg	SC	TNF	Rheumatoid arthritis, juvenile idiopathic arthritis, psoriatic arthritis, ankylosing spondylitis, Crohn’s disease, ulcerative colitis, plaque psoriasis, hidradenitis suppurativa, uveitis	2018
Ilumya	tildrakizumab-asmn	Sun Pharma Global	100 mg/mL	100 mg	SC	IL-23	Moderate-to-severe plaque psoriasis	2018
Takhzyro	lanadelumab-flyo	Dyax	150 mg/mL	300 and 150 mg	SC	Plasma kallikrein inhibitor	Hereditary angioderma	2018
Trogarzo	ibalizumab-uiyk	Theratechnologies	150 mg/mL	2000 and 800 mg	IV	CD4	HIV-1	2018
Ultomiris	ravulizumab-cwvz	Alexion	10 and 100 mg/mL	Between 300 and 3600 mg 300 mg for 5 to 10 kg, up to 3600 mg for 100 kg or more of body weight	IV	Complement	Paroxysmal nocturnal hemoglobinuria, atypical hemolytic uremic syndrome, generalized myasthenia gravis	2018
Beovu	brolucizumab-dbll	Novartis	120 mg/mL	6 mg	Intravitreal	VEGF	Neovascular (wet) age-related macular degeneration, diabetic macular edema	2019
Skyrizi	risankizumab-rzaa	Abbvie	150 mg/mL	150 mg	SC	IL-23	Plaque psoriasis, psoriatic arthritis	2019
Darzalex faspro	Daratumumab, hyaluronidase-fihj	Janssen	120 mg and 2000 U hyaluronidase/mL	1800 mg and 30,000 U hyaluronidase	SC	CD38	Multiple myeloma	2020
Enspryng	satralizumab-mwge	Genentech	120 mg/mL	120 mg	SC	IL-6	Neuromyelitis optica spectrum disorder	2020
Phesgo	pertuzumab, trastuzumab, hyaluronidase-zzxf	Genentech	80 mg, 40 mg, and 2000 U/mL; 60 mg, 60 mg, and 2000 U/mL	1200/600 mg and 600/600 mg (pertuzumab/ trastuzumab)	SC	HER2	HER2-positive metastatic breast cancer	2020
Vyepti	eptimezumab-jjmr	Lundbeck Seattle Biopharmaceuticals	100 mg/mL	100 mg	IV	Calcitonin	Migraine	2020
Adbry	tralokinumab-ldrm	AS Pharma	150 mg/mL	600 and 300 mg	SC	IL-13	Atopic dermatitis	2021
Aduhelm	aducanumab-avwa	Biogen	100 mg/mL	10 mg/kg	IV	Amyloid beta	Alzheimer disease	2021
Evkeeza	evinacumab-dgnb	Regeneron	150 mg/mL	15 mg/kg	IV	ANGPTL3	Homozygous familial hypercholesterolemia	2021
Saphnelo	anifrolumab-fnia	AstraZeneca	150 mg/mL	300 mg	IV	IFN	Systemic lupus erythematosus	2021
Tezspire	tezepelumab-ekko	AstraZeneca	110 mg/mL	210 mg	SC	TSLP	Severe asthma	2021
Vabysmo	faricimab-svoa	Genentech	120 mg/mL	6 mg	Intravitreal	VEGF, ANG2	Neovascular (wet) age-related macular degeneration, diabetic macular edema	2022
Beyfortus	nirsevimab-alip	AstraZeneca	100 mg/mL	50, 100, and 200 mg	IM	RSV F protein	Neonates and children with RSV	2023
Bimzelx	bimekizumab-bkzx	UCB	160 mg/mL	320 mg	SC	IL-17A, IL-17F	Plaque psoriasis	2023
Leqembi	lecanemab-irmb	Eisai	100 mg/mL	10 mg/kg	IV	Amyloid beta	Alzheimer disease	2023
Omvoh	mirikizumab-mrkz	Eli Lilly	20 mg/mL 100 mg/mL	300 and 200 mg	IV or SC	IL-23	Active ulcerative colitis	2023
Rystiggo	rozanolixizumab-noli	UCB	140 mg/mL	420, 560, and 840 mg	SC	FcRn	Myasthenia gravis	2023
Veopoz	pozelimab-bbfg	Regeneron	200 mg/mL	30 and 10 mg/kg	IV or SC	C5	Complement hyperactivation, angiopathic thrombosis, protein-losing enteropathy	2023
Yuflyma	Biosimilar (adalimumab-aaty)	Celltrion	100 mg/mL	40, 80, and 160 mg	SC	TNF	Rheumatoid arthritis, juvenile idiopathic arthritis, psoriatic arthritis, ankylosing spondylitis, Crohn’s disease, ulcerative colitis, plaque psoriasis, hidradenitis suppurativa	2023
Zymfentra	infliximab-dyyb	Celltrion	120 mg/mL	120 mg	SC	TNF	Active ulcerative colitis, Crohn’s disease	2023

*ANG2*, angiopoietin 2; *ANGPTL3*, angiopoietin-like protein 3; *FcRn*, neonatal crystallizable fragment receptor; *IgE*, immunoglobulin E; *IL-1β, −2, −4, *etc*.*, interleukin −1β, −2, −4, etc.; *IM*, intramuscular; *PCSK9*, proprotein convertase subtilisin/kexin type 9; *RSV*, respiratory syncytial virus; *TNF*, tumor necrosis factor; *TSLP*, thymic stromal lymphopoietin; *VEGF*, vascular endothelial growth factor

To date, only four mAbs in cancer therapy have transitioned to SC administration (PharmaCircle.com (PharmaCircle.com n.d.)). These include Darzalex Faspro (daratumumab; Janssen) for multiple myeloma, Herceptin Hylecta (trastuzumab and hyaluronidase-oysk; Genentech) for human epidermal growth factor receptor 2 (HER2)–overexpressing breast cancer, Rituxan Hycela (rituximab and hyaluronidase human; Genentech) for chronic lymphocytic leukemia and non-Hodgkin lymphoma, and Phesgo (pertuzumab, trastuzumab, and hyaluronidase-zzxf; Genentech) for HER2-positive breast cancer. Merck is actively developing an SC formulation of Keytruda (pembrolizumab) (Taylor 2023). The four products approved by the FDA for oncology treatment have a concentration of 120 mg/mL, are administered in volumes of 5–15 mL, and are co-formulated with recombinant hyaluronidase to facilitate the delivery of large volumes. These formulations, however, depend significantly on larger administration volumes and the potential requirement for recombinant hyaluronidase.

## Challenges in the development of high-concentration antibody products

The solution behavior and stability of antibody molecules rely predominantly on intermolecular interactions involving primarily the protein component, or protein–protein interactions (PPIs). These interactions are influenced by the specific sequence of amino acids and their three-dimensional folded structure, as well as the solution environment in which the protein resides. The carbohydrate component in antibodies can also contribute to interactive effects (Abu Hammad et al. 2023; Jefferis 2009). This environment comprises elements such as pH, temperature, buffer composition and the presence of specific excipients (Pindrus et al. 2015; Baek and Zydney 2018). At high concentrations, the spacing between antibody molecules decreases, which increases the likelihood of inter-molecular interactions (Raut and Kalonia 2015). This in turn may cause reversible self-association or even irreversible aggregation (Kollar et al. 2020), which can negatively affect the antibody’s activity and physical stability (Raut and Kalonia 2015).

**Fig. 4:**
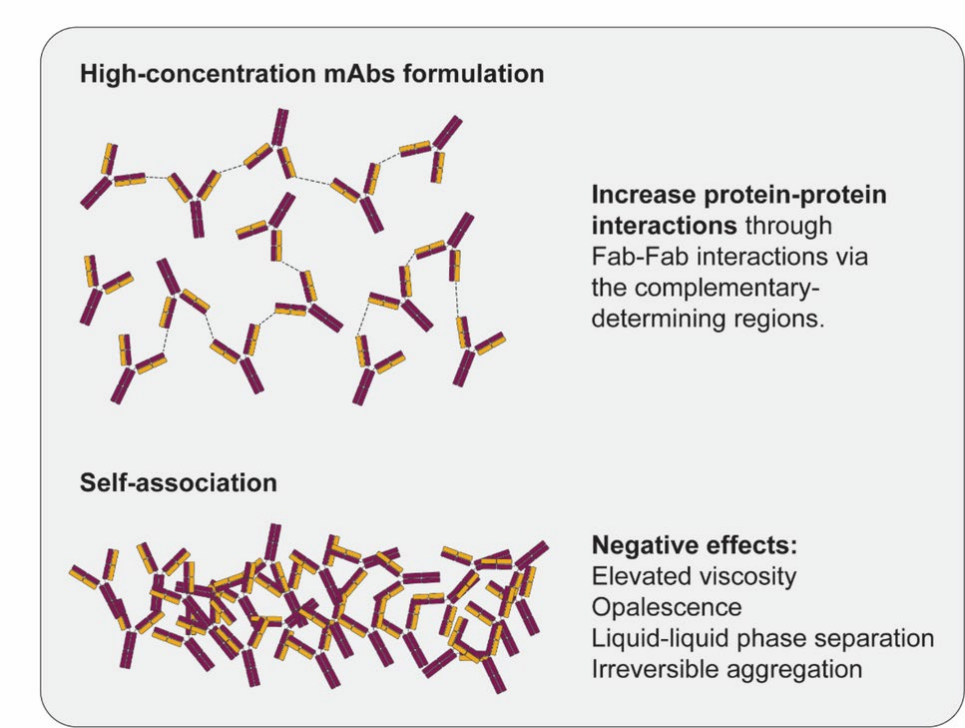
mAb self-association under high-concentration conditions and their negative effects

Antibody aggregation can occur through either covalent bond formation, such as intermolecular disulfide linkages, or noncovalent associations involving mainly hydrophobic and/or electrostatic interactions (Baek and Zydney 2018). In high-concentration antibody solutions, self-association occurs mainly through interactions between fragment antigen-binding antibodies via complementary-determining regions (Fig. 4) (Kollar et al. 2020).

As the concentration of antibody in solution increases, the viscosity of the solution can greatly increase. This escalation is driven by the increasing disruption of the laminar streamline flow of the solution. Concomitant with this is a decrease in distance between antibody molecules, which can facilitate noncovalent interactions, including van der Waals forces, hydrogen bonds, dipole–dipole interactions, and hydrophobic interactions. It can also produce associations of antibody molecules, which can lead to a further increase in the viscosity, depending on the conformation of the association complexes (Zidar et al. 2020; Harding 1997; Lu et al. 2006).

**Fig. 5:**
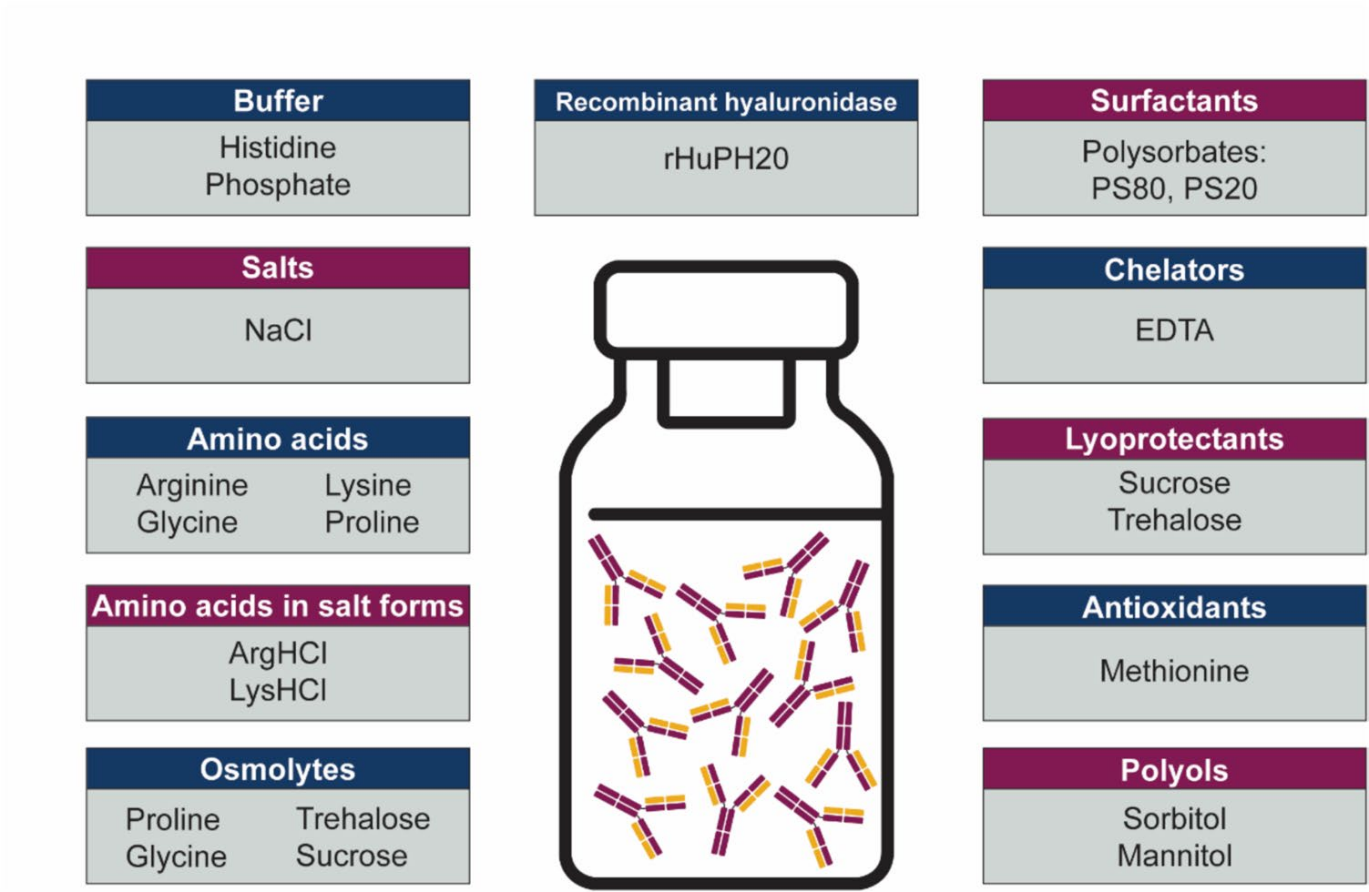
Commonly employed excipients in HCmAP formulations

The production of highly concentrated mAbs is often linked to difficulties in manufacturing, storage, shipping, and administration. This is primarily due to elevated viscosity and opalescence, as well as a greater propensity for aggregation resulting from intensified molecular interactions (Kollar et al. 2020). To address these challenges, extensive research has been conducted over the past decade to identify the most suitable formulation excipients to minimize the extent of molecular interactions. Table 2 provides a summary of the challenges, impacts, and formulation strategies (Fig. 5) reported in the development of high-concentration mAbs.

**Table 2:** Challenges, impacts, and formulation strategies in the development of HCmAP

Stage	Challenges	Impact	Formulation strategies
Manufacture	**High viscosity:** UF/DF: raise pump’s back-pressure while reducing transmembrane flux (Garidel et al. 2017 ) Clogging of UF/DF membrane (Desai et al. 2023 ) UF/DF is a possible cause of aggregation **Sterile filtration:** Low filter fluxes (Rodrigues et al. 2021 ) **Filling process:** Challenges with pump operation (Garidel et al. 2017 )	Increased processing time for restriction of product flow (Kollar et al. 2020 ) Increased manufacturing cost (Tomar et al. 2016 ) (product loss) Destabilization of drug substance (Tomar et al. 2016 ) Drying of product and blockage of needles (Garidel et al. 2017 )	**Viscosity-lowering excipients:** Inorganic salts: NaCl, NaSCN, Na_2_SO_4_ (Dear et al. 2017 ) Amino acids: arginine, proline, glycine, methionine (Ghosh et al. 2023; Kollar et al. 2020; Desai et al. 2023 ) Amino acids in salt forms: ArgHCl, LysHCl, HisHCl (Strickley and Lambert 2021; Rodrigues et al. 2021; Kemter et al. 2018 ) Caffeine (Zeng et al. 2021 ) Organic acid: DPA, diethanolamine-DPA, ethanolamine-DPA (Ke et al. 2018 ) Buffer: histidine, acetate, citrate, phosphate (Jiskoot et al. 2022; Kollar et al. 2020; Ghosh et al. 2023; Kemter et al. 2018 ) **Decrease aggregation:** Osmolytes: sucrose, proline, glycine, trehalose (Hung et al. 2018 ), alanine (Dear et al. 2017 ) Amino acids: arginine, arginine glutamate (Desai et al. 2023; Srivastava et al. 2022 )
Storage and shipping	**Opalescence:** Turbidity in a solution might serve as an early sign of aggregation **Liquid–liquid phase separation**: Forming two phases, protein-poor and protein-rich **Aggregation**	Patient concerns about the safety of the product’s formulation Reduced product stability or quality Reduced product stability and biological activity. Possible toxicity issues (see also below)	**Stabilizing effects:** **Surfactants:** Polysorbates like PS80 and PS20 (Baek and Zydney 2018; Holstein et al. 2020 ) **Metal chelator:** EDTA (Holstein et al. 2020 ) In downstream purification processes, focus on eliminating troublesome lipases or employ a knockout approach to remove them upstream (Holstein et al. 2020 ) Minimize light exposure (Holstein et al. 2020 ) Decrease areas air/liquid interface by, e.g., reducing headspace **Lyoprotectants:** Sucrose, trehalose, mannitol, sorbitol (Kollar et al. 2020; Batens et al. 2018; Strickley and Lambert 2021 ) **Antioxidants:** Methionine(Ghosh et al. 2023; Fevre et al. 2022 ) Storage at refrigerated conditions and product-specific stable formulations(Desai et al. 2023 )
Delivery	**High viscosity:** Increased tissue back-pressure Increased injection time Greater adherence of the viscous solution to product container surfaces **Aggregation**	Injection pain Decreased patient compliance (Kollar et al. 2020 ) Unrecoverable product losses in the primary container Aggregation can impact biological activity and potency (Jiskoot et al. 2022; Bansal et al. 2020 )	Advanced drug delivery technologies: autoinjectors, pre-filled injectors Use of rHuPH20 for larger-volume injection (Jiskoot et al. 2022; Kollar et al. 2020; Wang et al. 2021 ) Applying specific surface coating to limit adsorption of proteins on the internal glass wall **Tonicity agents:** disaccharides (sucrose, trehalose), polyols (sorbitol, mannitol), sodium chloride(Falconer 2019; Ghosh et al. 2023 )

*DPA*, dipicolinic acid; *rHuPH20*, recombinant human hyaluronidase

## Manufacturing challenges

Toward the end of the manufacturing process, a step involving ultrafiltration/diafiltration (UF/DF) is included for concentrating proteins (Garidel et al. 2017; Baek and Zydney 2018), followed by a final step of sterile filtration. The increase in viscosity and also possible self-associative/aggregative effects with concentration is a problem not only for the patient but also for manufacturing (Holstein et al. 2020; Zhang and Liu 2017) and leads to various challenges. Elevated viscosity during UF/DF can lead to increased pump back-pressure and a reduction in transmembrane flux and/or filtration flow (Garidel et al. 2017), potentially resulting in prolonged processing time, destabilization of the drug substance, and increased manufacturing expenses (Tomar et al. 2016). Other process steps, such as sterile filtration, can be hindered by filter membrane clogging (Rodrigues et al. 2021) and filling that might also be affected by highly viscous antibody solutions (Garidel et al. 2017).

One of the objectives in developing successful formulations of highly concentrated mAbs is to reduce nonspecific PPIs and carbohydrate interactions by using electrostatic and hydrophobic interactions to both disrupt protein self-association and decrease viscosity (Jiskoot et al. 2022). Excipients for viscosity reduction include salts such as sodium chloride (NaCl), which interact with charged residues on the protein surface. NaCl lowers viscosity by providing charge shielding, which in turn reduces electrostatic interactions among protein molecules (Rodrigues et al. 2021). Amino acids such as glycine, lysine, proline, and histidine (Jiskoot et al. 2022) can decrease viscosity by influencing electrostatic and nonpolar interactions (Rodrigues et al. 2021). Arginine is commonly employed to reduce viscosity and aggregation and is the most frequently used additive (Srivastava et al. 2022). Amino acids in their salt forms, such as arginine monohydrochloride (Arg-HCl), decrease both PPI and viscosity by more complex mechanisms. As a salt, Arg-HCl can affect viscosity by charge shielding in the same way as NaCl. It can also decrease hydrophobic interactions by engaging with aromatic and charged residues of proteins (Zeng et al. 2021). To mitigate hydrophobic interactions, antibodies must be stabilized in their natural folded state at high concentrations through the use of osmolyte depletants such as polymers and polysaccharides, proline, glycine, and trehalose (Hung et al. 2018). Hung et al. (2018) have claimed that proline reduced the viscosity of a 225-mg/mL solution of mAb by up to threefold, lowering it to 25 centipoise (cP) at pH 6.0.

Formulation pH has a significant impact on the physical stability of antibodies by influencing the number and distribution of charges on the protein surface. Formulation pH plays a critical role in chemical stability by controlling various degradation pathways such as disulfide bond formation, deamidation, and fragmentation. Selecting an appropriate buffering agent can potentially achieve both pH control and antibody stabilization (Wang et al. 2007). Protein formulations typically use buffers such as histidine, acetate, or citrate to maintain the solution pH within a range of 3–7. Of these buffers, histidine is one of the most widely used amino acid buffers in highly concentrated formulations (Ghosh et al. 2023).

In the pursuit of novel excipients that are capable of enhancing the manufacturing process for highly concentrated proteins, Ke et al. (2018) explored the organic acid dipicolinic acid (DPA), focusing specifically on ethanolamine-DPA. Their results showed a significant reduction in viscosity (cP) for multiple mAbs (mAb1, mAb2, mAb3, and mAb4) of up to 22, 9, 14, and 17 cP, respectively, compared with the control, resulting in respective viscosities of 382, 98, 706, and 158 cP at a concentration of approximately 160 mg/mL. The results of this exploration highlight the potential of DPA salts in highly concentrated formulations, offering a potential alternative for formulation scientists during the selection of excipients during formulation development.

Caffeine has demonstrated promise as an excipient for reducing viscosity without compromising protein stability or potency. Zeng et al. (2021) investigated the impact of caffeine on viscosity reduction in HCmAP by using ipilimumab at a concentration of 200 mg/mL and demonstrated that caffeine is capable of decreasing viscosity in different formulation buffers. When phosphate-buffered saline was used as a buffer, the control viscosity was 76 cP; after the addition of 75 mM caffeine, the viscosity was reduced to 17 cP, a 78% decrease. Similarly, with the commercial formulation vehicle, the control viscosity was 37 cP, and the addition of 75 mM caffeine reduced it to 16 cP, which was 57% lower than the control (Zeng et al. 2021).

## Storage and shipping challenges

Opalescence and liquid–liquid phase separation may arise in highly concentrated proteins, presenting challenges during storage, particularly at refrigerated temperatures. In addition to affecting the visual appearance of the formulation, opalescence can indicate the presence of aggregates in the solution induced by temperature, light exposure, or agitation during shipping or as a precursor to phase separation, resulting in decreased product stability. In liquid–liquid phase separation, the initially homogeneous protein solution is separated into two distinct liquid phases, resulting in a region with lower protein content and a region with higher protein content (Raut and Kalonia 2015; Kollar et al. 2020).

The addition of surfactants, mainly polysorbates, is used to lower the interfacial concentration of proteins to substantially reduce shipping-induced aggregation (Baek and Zydney 2018). However, polysorbates are susceptible to degradation caused by autoxidation due to factors such as temperature, light, and oxidants (including metals). They are also prone to hydrolysis, mainly through enzymatic pathways (Jiskoot et al. 2022). Residual host cell proteins that can co-purify with protein products, such as lipases, have been seen to increase the degradation of polysorbate 80. To mitigate lipase activity, it is possible to incorporate a metal chelator such as ethylenediaminetretraacetic acid (EDTA) into the formulation. Because lipases rely on calcium for their activity, chelating calcium may serve as an effective control strategy (Holstein et al. 2020).

Highly concentrated proteins encounter storage conditions that may lead to oxidation, affecting their functionality, aggregation, folding state, and solubility. Methionine is often included in therapeutic formulations as an antioxidant (Fevre et al. 2022). Chelators (EDTA) also play a crucial role in reducing oxidation by effectively capturing free metal ions such as copper (from raw materials as a trace contaminant) before these ions can initiate protein oxidation (Brovč et al. 2020).

## Delivery challenges

The high viscosity of high-concentration biologics is an area of significant concern. The viscosity of a formulation significantly influences the force required for injection, an effect that becomes particularly pronounced when using narrow-gauge needles, such as commonly used 26G or 27G gauge needles (Marschall et al. 2023). The increased pressure required to inject the drug results in slower injection rates. In addition, tissue back-pressure is increased when a highly viscous formulation is injected into the body (Kollar et al. 2020) because it encounters greater resistance in the tissues.

To facilitate self-administration through a thin needle, the viscosity of an HCmAP for SC delivery should be less than 20 cP (Baek and Zydney 2018). The previously discussed formulation strategies to reduce viscosity play a critical role in addressing the challenges associated with drug delivery. An alternative approach to delivering large doses without concentrating the drug product is to increase the volume being administered.

Administration of large volumes in SC tissue faces challenges, however, due to the presence of hyaluronan, a polysaccharide component of the extracellular matrix that acts as a physical barrier. Hyaluronan prevents bulk fluid flow, resulting in prolonged infusion times and limiting drug dispersion and absorption (Knowles et al. 2021). This constraint affects not only the delivery rate of medications but also overall patient experience, often resulting in discomfort, erythema, and reduced adherence to treatment regimens (Dolton et al. 2021). To address this issue, recombinant human hyaluronidase, an enzyme that degrades hyaluronan in the skin, can be incorporated into the formulation with the protein solution and co-administered. By effectively reducing the viscosity of the extracellular matrix, hyaluronidase dramatically enhances tissue permeability. This allows for the rapid delivery of large dose volumes and improves patient tolerability (Connor et al. 2023). Table 3 compares the advantages and disadvantages of IV administration with SC administration and SC administration with hyaluronidase.

**Table 3:** Advantages and disadvantages of antibody therapeutic delivery via IV and SC administration with hyaluronidase

Criterion	IV administration	SC administration	SC administration with hyaluronidase
**Advantages**			
Rapid onset	Delivers drugs directly into the bloodstream for rapid action	Slower onset than IV	Enhanced absorption speeds up onset compared with standard SC
Complete bioavailability	Achieves 100% bioavailability as it enters directly into circulation	Lower systemic absorption initially	Increases tissue permeability for improved drug absorption
Controlled dosing	Allows precise control over dosing and rate of administration	May have variable absorption rates	Allows larger volumes with more consistent absorption rates
Suitable for large-volume doses	Can accommodate larger fluid volumes	Limitations on volume per injection	Facilitates larger volume SC administration, suitable for biologics
**Disadvantages**			
Requires clinical setting	Typically needs health care professional and clinical settings	Can facilitate home administration (NB: supervised where appropriate)	Can facilitate home administration (NB: supervised where appropriate)
Increased risk of complications	Higher risk of infection, phlebitis, and complications from catheter placement	Lower risk of systemic complications	May cause local reactions due to enzymatic activity
Patient discomfort	More invasive and potentially unpleasant for patients	Generally less invasive and painful	Slightly increased possibility of local tissue reactions
Slower onset	Not applicable for IV—rapid by nature	Slower onset is a disadvantage for acute needs	Quicker than standard SC to address acute issues effectively
Limited volume	Not applicable for IV—high-volume capability	Limits ability to deliver larger drug dosages	Increased capacity to accommodate larger dosages
Compatibility with formulations	Compatible broadly with various IV preparations	Limited by drug formulations suitable for SC	Must ensure drug compatibility with hyaluronidase

## Move toward HCmAP

### Assessment of treatment delays in immunotherapy during the COVID-19 pandemic

The studies analyzed revealed significant treatment delays during the COVID-19 pandemic. In China, Sha et al. (2020) highlighted a notable increase (29%) in treatment delays for lung cancer patients during the pandemic compared with the pre-pandemic period (< 5%). Similarly, a study by Zhai et al. (2023) identified increased rates (4%) of delays in immunotherapy administrations and a higher proportion of patients experiencing delays in receiving treatment from February to March 2020 (early COVID-19 period) in comparison with the period from February to March 2019 (pre-COVID). In the UK, Pala et al. (2020) found that treatment was delayed by an average of 4 weeks, ranging from 3 to 9 weeks. In a prospective cohort study in Germany and the UK, De Souza et al. (2023) identified delays in treatment for hepatocellular cancer with a median time to treatment of 1.6 months. In China, Chen et al. (2020) found an average treatment delay of 3.8 months.

### Factors contributing to delays in immunotherapy and mitigation strategies

The main factors contributing to treatment delays have been categorized into three groups: provider related, patient related, and context related. Provider-related factors include the closure of cancer care facilities due to lack of personnel and shortage of personal protective equipment (Pareek et al. 2021), rigorous screening and admission criteria (Sha et al. 2020), and prioritizing the enhancement of care for COVID-19 patients (De Souza et al. 2023). Patient-related factors include patients’ fear of acquiring COVID-19 during hospital visits, financial issues (Pareek et al. 2021), limited familial support or caregivers (Sha et al. 2020), and fewer visits by patients with elevated risk factors such as advanced age and comorbidities (Pala et al. 2020). Context-related factors include quarantine and lockdown measures to control the spread of the virus, as well as lack of public transport services (Pareek et al. 2021).

We observed a trend in immunotherapy during the lockdown period, which was reported in three articles (Pareek et al. 2021; Zhai et al. 2023; Russell et al. 2022). Immunotherapy was increasingly preferred over chemotherapy, which is considered a myelosuppressive therapy. Immunotherapy was also identified as the oncology treatment with the most frequent adjustments (Sha et al. 2020). These adjustments involved extending the intervals between immunotherapy treatments to reduce the frequency and duration of clinical visits (Ottaviano et al. 2020) through the administration of higher concentrations of the drug. For example, some studies reported switching pembrolizumab administration from 200 mg every 3 weeks to 400 mg every 6 weeks (Zhai et al. 2023; Aguiar-Ibanez et al. 2023) or adjusting durvalumab administration from every 2 weeks to every 4 weeks (Joshi et al. 2021).

### Consequences of delays in immunotherapy administration

Despite the clear increase in treatment delays during the pandemic, the disease of cancer patients was reported to be under control (Pala et al. 2020). A multicenter cohort study found no significant impact of delayed treatment on progression-free survival (De Souza et al. 2023).

Nowara et al. (2021) and Meng et al. (2020) observed that cancer patients who contracted COVID-19 exhibited a higher fatality rate than COVID-19 patients without cancer. This was also explored in a UK study by Lee et al. (2020), who evaluated and compared adult patients with cancer with and without COVID-19. It was reported that the susceptibility to COVID-19 among cancer patients differed depending on the type of tumor. Age and sex were significant factors; older patients and those with hematological malignancies faced a particularly high risk. Not all cancer patients were equally affected.

The analysis of treatment delays in immunotherapy during the COVID-19 pandemic reveals significant disruptions in the administration of cancer treatments stemming from a multitude of factors, including those associated with health care providers, patients, and contextual circumstances. The use of HCmAP presents a promising avenue in addressing these challenges. Enabling supervised administration via SC injection at home could significantly mitigate the impact of treatment delays, ensuring uninterrupted treatment even during disruptions to hospital-based health care services and leading to improved treatment experiences and patient compliance. Roche recently announced that the European Medicines Agency has issued a positive opinion recommending an update to the European Union label for Phesgo for HER2-positive breast cancer. If approved and safely established in a clinical setting, it will be possible for health care professionals to administer Phesgo outside of a clinical setting (such as in a person’s home).

## Challenges to and strategies for developing HCmAP

Transitioning from IV infusion to SC injection to allow at-home administration (at-home supervised administration), using hand-held devices such as prefilled syringes or autoinjectors, necessitates the development of HCmAP. This need is primarily due to the constrained volume capacity (< 3 mL) of hand-held SC injections (Badkar et al. 2021). Garidel et al. (2017) determined the potential maximum concentration of mAbs by establishing a theoretical upper limit. This estimation relied on three-dimensional structures and geometric factors, considering the packing of molecules with shapes like spheres, rectangles, and truncated cones. The concentration was calculated by using the immunoglobulin G1 mAb model, and the empirical data supported a recommendation to set the limit at levels of < 500 mg/mL.

An innovative approach that may be considered in the future for delivery of high-dose biologics has recently been developed by Elektrofi, utilizing non-aqueous microparticle suspensions generated to achieve ultra-high protein concentrations (up to 500 mg/mL) (Shadbar et al. 2025). This method enables low-volume subcutaneous injections, deliverable in under 20 s with injection forces below 20 N, while demonstrating bioequivalence to aqueous formulations in rodent models (Shadbar et al. 2025).

In an alternate approach, Marschall et al. (2023) investigated protein powder suspensions in non-aqueous vehicles, prepared by using spray-dried powders, to explore their potential to lower viscosity. This approach provides an alternative that could potentially lead to lower viscosity than that of equivalent aqueous solutions, thereby addressing the challenge of formulating concentrations exceeding 200 mg/mL. When injected, these formulations exhibit a shear thinning viscosity of < 10 cP, minimizing the force required for injection. Nevertheless, multiple obstacles must be overcome before such a product is ready for the market. These challenges include a complex manufacturing process and the need for approval as a novel vehicle in biologic formulations by the FDA.

The various excipients proposed for reducing viscosity are typically incorporated during the later stages of drug development. However, finding an effective excipient strategy for highly concentrated mAb products is not always feasible. Currently emerging approaches complement excipient strategies and are implemented at an early stage during drug discovery. These methods employ computational techniques to identify antibody variants with reduced aggregation tendencies and greater colloidal stability. Molecular design also plays a pivotal role, as modifications to amino acid sequences may optimize mAb characteristics and minimize aggregation propensity (Dobson et al. 2016). By combining early-stage engineering of molecules with the use of formulation excipients, future endeavors may enable the manufacturing of HCmAP for immunotherapy, potentially facilitating at-home administration (supervised at-home administration).

### Sustainable and cost-effective solutions: HCmAP and SC administration

The goal of sustainability in health care is to minimize waste, carbon emissions, and environmental impacts (Javaid et al. 2022), ultimately aiming to enhance the quality, safety and value (Sherman et al. 2020) of pharmaceuticals. Health care services in the UK generate approximately 5% of the nation’s total CO_2_ emissions. The majority of these emissions originate from the supply chain, particularly in the form of medicines and equipment (Cussans et al. 2021). Similarly, the health care sector in the USA contributes to 9% of the six “criteria air pollutants” (those identified by the Environmental Protection Agency as requiring control to meet air quality standards), namely, ozone, particulate matter, carbon monoxide, lead, sulfur dioxide, nitrogen dioxide, and 9–10% of national greenhouse gas emissions (Sherman et al. 2020).

**Fig. 6:**
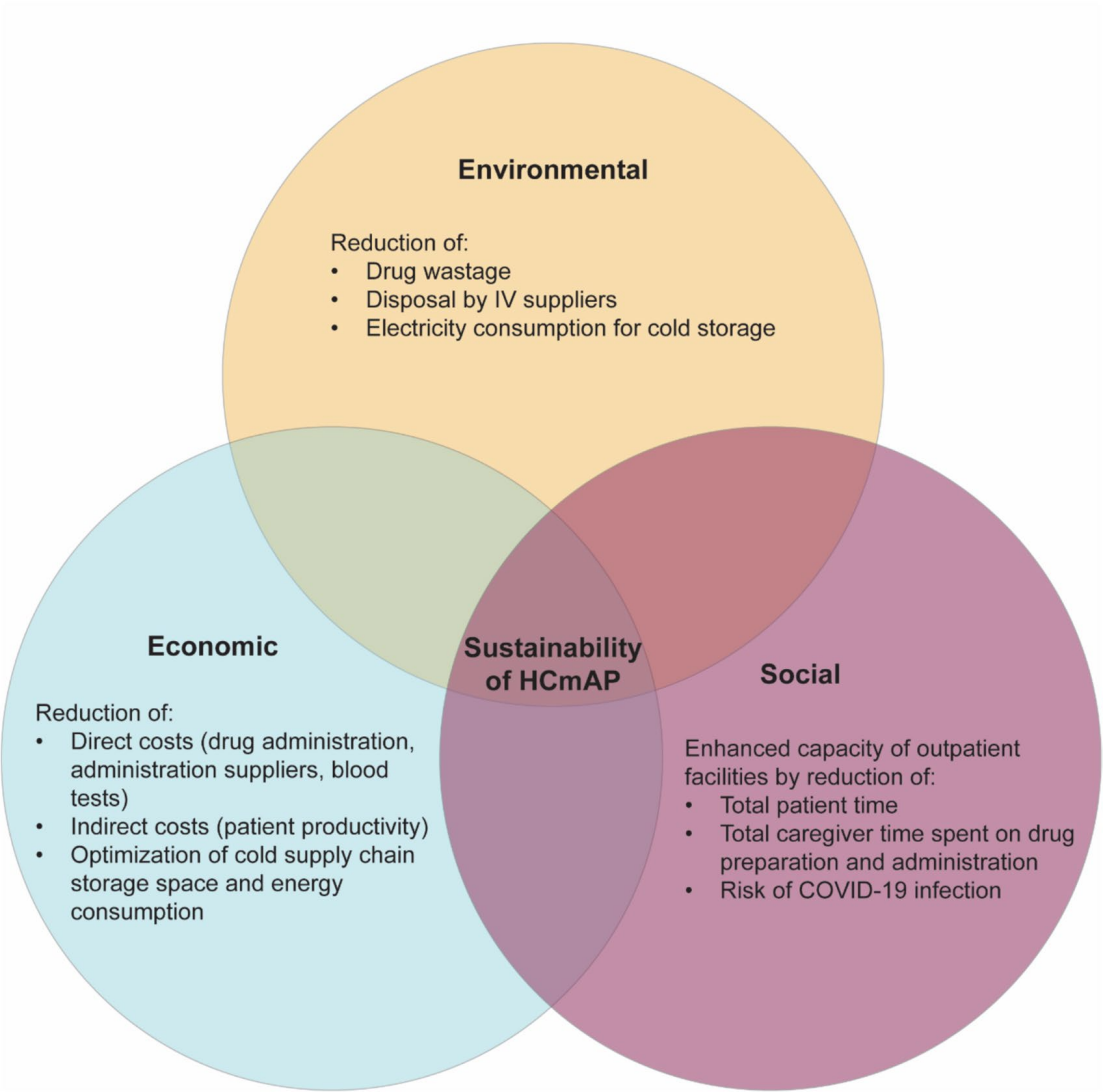
Multidimensional benefits of transitioning from IV to SC administration of HCmAP

Upon analyzing the transition from IV to SC administration facilitated by the development of HCmAP and innovative delivery technologies, we identified numerous reported advantages. These benefits extend beyond chronic diseases to include oncology treatment, indicating potential improvements in the sustainability of future drug products (Fig. 6).

SC administration of HCmAP also has benefits from an economic perspective. Decreases in both direct and indirect costs have been documented. Direct costs include expenses incurred in the delivery of the drug, such as administration fees, health care workers’ salaries, and supplier costs. Indirect costs involve factors like lower patient productivity in the workforce and the need for a broader range of health care services, such as those incurred by the potential for acquiring COVID-19 infection during hospital stays or via public transportation (Heald et al. 2021). The costs associated with drug wastage also represent a significant financial burden (Tjalma et al. 2018).

Anderson et al. (2019) published evidence to evaluate the economic consequences of SC versus IV administration of approved cancer therapies. Most of the studies focusing on economic outcomes favored SC administration. The noteworthy reduction in the time required for preparation, administration, and post-administration care is reported to have a significant impact on costs. McCloskey et al. (2023) reported that the direct costs for SC administration were roughly 1–6% less than those linked to IV administration.

The most effective approach to mitigate the presence of pharmaceuticals in the environment is to reduce the quantity of unused medication (Lipkin 2012). Ponzetti et al. (2016) analyzed the comparative impact of SC versus IV administration of rituximab and trastuzumab in Italy. They identified potential reductions in drug wastage with SC therapy due to its fixed-dose format. Specifically, for trastuzumab, the total median annual wastage for a complete treatment was calculated as 7376 mg for IV administration and 0 mg for the SC route. The wastage of drugs impacts costs for both patients and health care systems (Lien et al. 2016) and also has environmental repercussions (Smale et al. 2021).

In addition to these benefits, SC administration can also have reduced environmental impacts because the suppliers and devices needed for IV administration are not essential for SC administration. Ponzetti et al. (2016) observed a significant (54%) reduction in materials provided by medical staff. IV devices such as fluid bags and peripheral and central catheters often contain polyvinyl chloride and are disposed of in red bags for incineration. The combustion of polyvinyl chloride leads to the formation of dioxins, which are carcinogenic and bioaccumulative (Lipkin 2012).

Switching to SC administration can also have cost and environmental impacts by reducing the amount of space needed for cold storage of the drug product, thereby also decreasing electricity consumption. Storage of mAb drug products is energy intensive, as they must often be stored at a temperature of 2–8 °C (Laptos and Omersel 2018). For example, IV daratumumab is available in two vials, 100 mg/5 mL and 400 mg/20 mL. A patient with a body weight of 70 kg would require 2.8 vials of the 400 mg/20 mL dose. In contrast, daratumumab formulated for SC administration comes in a single vial containing 1800 mg of daratumumab and 30,000 U of hyaluronidase per 15 mL. At the recommended fixed dose of 1800 mg, only one vial would be required for each patient. Over a 1-year treatment period, IV administration requires significantly more vials than the SC route. Increasing the adoption of SC administration leads to a significant reduction in the volume required for storage and shipping and therefore represents a step toward the establishment of a more sustainable supply chain.

The SC administration route can also contribute to social equity. SC administration is four times more time efficient than the IV route (Tjalma et al. 2018), allowing patients to spend less time in oncology day care, thereby reducing the time nurses spend per patient and expanding the opportunity to provide services to a greater number of patients (Tjalma et al. 2018; Tomarchio et al. 2017).

## Conclusion

The COVID-19 pandemic has further revealed the importance of innovative strategies for the administration of cancer therapies that provide more patient-convenient alternatives. SC immunotherapy administration offers numerous advantages over the IV route, most notably, patient compliance and satisfaction if the possibility of at-home administration becomes a reality. For oncology drugs, this would require clinical supervision and/or home visits, thereby facilitating the treatment of patients who cannot travel. In many cases, however, this transition will require the development of HCmAP because of the limited volume that can be administered via SC injection. The manufacturing process for HCmAP doses poses challenges, mainly from increased viscosity and physical instability of the formulation, which can be ameliorated by the use of specific excipients. The most commonly used excipients for these purposes are salts, such as NaCl, and amino acids, such as arginine. However, there are limitations in the extent to which viscosity can be reduced while maintaining satisfactory protein stability.

Transitioning to SC administration of cancer therapies also offers a cost-effective approach, primarily through reduced preparation and administration times. SC administration benefits the environment by reducing the drug wastage and greenhouse gas emissions that are associated with fewer hospital visits. Social benefits include improved patient convenience and quality of life. Finally, SC administration of biopharmaceutical formulations can enable health care facilities to accommodate more patients, thereby promoting equitable access to care.

## Data Availability

No datasets were generated or analysed during the current study.
